# Crosstalk between Inflammation and ROCK/MLCK Signaling Pathways in Gastrointestinal Disorders with Intestinal Hyperpermeability

**DOI:** 10.1155/2016/7374197

**Published:** 2016-09-26

**Authors:** Lijun Du, John J. Kim, Jinhua Shen, Ning Dai

**Affiliations:** ^1^Department of Gastroenterology, Sir Run Run Shaw Hospital, School of Medicine, Zhejiang University, Hangzhou, Zhejiang Province, China; ^2^Division of Gastroenterology, Loma Linda University, Loma Linda, CA, USA; ^3^Department of Gastroenterology, Affiliated Hospital of Shaoxing University, Shaoxing, Zhejiang Province, China

## Abstract

The barrier function of the intestine is essential for maintaining the normal homeostasis of the gut and mucosal immune system. Abnormalities in intestinal barrier function expressed by increased intestinal permeability have long been observed in various gastrointestinal disorders such as Crohn's disease (CD), ulcerative colitis (UC), celiac disease, and irritable bowel syndrome (IBS). Imbalance of metabolizing junction proteins and mucosal inflammation contributes to intestinal hyperpermeability. Emerging studies exploring* in vitro* and* in vivo* model system demonstrate that Rho-associated coiled-coil containing protein kinase- (ROCK-) and myosin light chain kinase- (MLCK-) mediated pathways are involved in the regulation of intestinal permeability. With this perspective, we aim to summarize the current state of knowledge regarding the role of inflammation and ROCK-/MLCK-mediated pathways leading to intestinal hyperpermeability in gastrointestinal disorders. In the near future, it may be possible to specifically target these specific pathways to develop novel therapies for gastrointestinal disorders associated with increased gut permeability.

## 1. Introduction

Epithelium of the gastrointestinal tract forms a dynamic and selective barrier between the external and the internal environment. It enables the absorption of dietary nutrients and the restriction of potentially harmful compounds [1]. Under physiologic conditions, the passage of molecules occurs selectively across cellular sheets by transcellular transport or paracellular pathway dictated by both electrical charge and size [2].

The primary structure that regulates intestinal barrier is the apical junctional complex (AJC) which is located at the paracellular space and contributes to maintaining tissue integrity and cell-to-cell communication [3, 4]. The major constituents of the AJC are the tight junction (TJ) and the subjacent adherens junction (AJ). TJ and AJ are closely positioned at the apical part of the lateral plasma membrane and are physically linked to the intracellular cytoskeleton. Both TJ and AJ are multiprotein complexes composed of transmembrane proteins (occludin, claudin family proteins, and E-cadherin) and cytoplasmic proteins (zonula occludens (ZO) family proteins and p120 catenin proteins) [5]. TJ and AJ cytoplasmic proteins have been shown to interact with the cytoskeleton [6]. Coupled together, the AJC regulates paracellular permeability under various luminal stimuli [7, 8]. The actomyosin cytoskeleton is critical for assembly, maintenance, and disassembly of the epithelial paracellular junction. Multiple* in vitro* and* in vivo* studies have demonstrated the role of nonmuscle myosin II (NM II) as a key regulator of intestinal epithelial junction and barrier integrity [8, 9]. The activation of actomyosin cytoskeleton is regulated by reversible forms between globular monomeric actin (G-actin) and filamentous actin (F-actin). F-actin associated NM II is an important regulator of highly flexible and adaptable actomyosin cytoskeleton. NM II is composed of two heavy chains, two regulatory light chains (RMLC), and two essential light chains [10]. The actin binding domain is located in the heavy chain and folded until RMLC is activated by phosphorylation, leading to contraction of actomyosin [11]. NM II is the principal cytoskeletal motor that mediates the static tension and contractility of actin filaments [10]. Multi kinases including Rho-associated coiled-coil containing protein kinase (ROCK), myosin light chain kinase (MLCK), citron kinase, and leucine zipper interacting kinase (ZIPK) can phosphorylate MLC of NM II [12–15]. However, only MLCK and ROCK have been implicated to be involved in TJ regulation during intestinal inflammation [16, 17]. In certain circumstances, the disruption of the intestinal barrier can be indicated by a decrease in transcellular electrical resistance (TER) and an increase in paracellular permeability. The compromised intestinal barrier dysfunction may be either causative or consequential. The disruption of the intestinal epithelial TJ barrier allows increased mucosal penetration of intestinal luminal toxic substances, pathogens, and antigens that can lead to intestinal mucosal injury and inflammation [18]. Proinflammatory cytokines like interferon (IFN)-*γ*, tumor necrosis factor (TNF)-*α*, interleukin- (IL-) 1*β*, IL-6, and IL-13 have been identified to contribute to the disruption of intestinal barrier [19–24]. Hyperpermeability and immune activation lead to a vicious self-propagating cycle. The components of the cycle including barrier dysfunction, abnormal immune response, and inflammatory stimuli may initiate and contribute to further epithelial barrier dysfunction and immune activation [25].

Previous studies have identified that intestinal hyperpermeability is an important pathogenic factor in a number of gastrointestinal diseases including ulcerative colitis (UC), Crohn's disease (CD), irritable bowel syndrome (IBS), and functional dyspepsia (FD) [26–29]. Therefore, the understanding of intracellular processes involved in the regulation of the intestinal epithelial TJ barrier function is important in developing therapeutic strategies to promote restoration of the intestinal TJ barrier in certain disease states [30, 31].

This review focuses on ROCK- and MLCK-mediated intracellular pathways which may play important roles in the regulation of internal cytoskeleton and are responsible for interaction between inflammation and increased intestinal permeability in specific gastrointestinal disorders.

## 2. ROCK Signaling Pathway

Rho proteins are members of the Ras superfamily of GTP-binding proteins (20- to 30-KDa), which have been shown to regulate a wide spectrum of cellular function [32]. As members of the Rho family, RhoA, RhoB and RhoC isoforms are regarded as core molecules that induce stress fibers to form and regulate cellular adherence by reconstructing cytoskeleton in response to extracellular stimuli [33]. Rho proteins function as a bimolecular switch by adopting different conformational states in response to the binding of GDP (inactive) or GTP (active). The GTP- and GDP-bound states are controlled primarily by two classes of regulatory molecules. GTPase-activating proteins (GAPs) increase the intrinsic rate of GTP hydrolysis, and guanine nucleotide-exchange factors (GEFs) facilitate the exchange of GDP for GTP [34]. GAPs and GEFs are highly expressed in epithelium and are activated by extracellular stimuli including inflammatory cytokines and bacterial products [35]. ROCKs are downstream effectors of the GTP-binding Rho proteins. The ROCKs consist of three major domains: RhoA binding domain (RBD), kinase domain that is responsible for catalytic activity, and cysteine-rich domain that is thought to participate in localization [36]. Two isoforms of ROCKs have been extensively studied: ROCKI and ROCKII. ROCKI is widely expressed in nonneuronal tissues, including heart, lung, and skeletal muscles. In contrast, ROCKII is mainly expressed in the brain [37]. ROCKs belong to the members of the serine/threonine protein kinases family that are characterized by their effect on the direct phosphorylation of MLC and inactivation of myosin–binding subunit of myosin phosphatase (MP). This leads to accumulation of phosphorylated myosin light chain (pMLC) and subsequent regulation of cytoskeletal contractility [36, 38, 39]. Furthermore, Rho-ROCK signaling activates LIM kinase and stabilizes actin filaments by inducing phosphorylation and inactivation of cofilin which is essential for actin filaments turnover. As a result, this mechanism contributes to spatial reorganization of actin cytoskeleton [40].

## 3. MLCK Signaling Pathway

MLCK is a Ca^2+^-calmodulin-dependent serine/threonine kinase that dynamically regulates actomyosin reorganization and cell contraction in both smooth-muscle and nonmuscle cells. The MLCK family is comprised of nonmuscle forms (210-KDa), short smooth-muscle forms (108-KDa), and telokin (21-KDa) that lacks enzymatic activity. Nonmuscle MLCK (nmMLCK) is predominantly expressed in epithelium, endothelium, and polymorphonuclear (PMN) leukocytes. Moreover, it has been identified that nmMLCK plays a vital role in modulating cell functions [41–43]. Previous studies showed that nmMLCK represents the principal MLCK in intestinal enterocytes and is responsible for Na^+^-nutrient cotransport-dependent TJ regulation [43]. NmMLCK phosphorylates MLC at threonine 18 and/or serine 19 leading to actin-myosin interaction and cytoskeletal sliding. This induces epithelial barrier breakdown [44]. MLCK is widely studied for its role on intestinal epithelial breakdown, which is critical for the pathogenesis of several diseases including burn injury, inflammatory bowel disease (IBD), and IBS [45–47]. In response to physiologic and pathophysiologic stimuli, MLCK-dependent regulation of epithelial barrier function may lead to increased intestinal permeability resulting from tight junction breakdown [30, 31]. Moreover, one study found that the magnitude of MLCK expression and presence of increased MLC phosphorylation strongly correlated with active inflammation [45]. Thus, targeted nmMLCK inhibition may be a potential target for epithelial restoration and inhibition of inflammatory diseases progression.

## 4. Crosstalk between Inflammation and MLCK and/or ROCK in Increased Intestinal Permeability

TJ activity is regulated by a wide variety of physiologic and pathologic conditions including those in the RhoA pathway and inflammatory cytokines [48, 49]. The activation of actomyosin contraction assessed by phosphorylation of MLC has been implicated in TJ regulation in the epithelium. TJ barrier can be regulated immediately by signal transduction cascades, which frequently require activation of MLCK and ROCK [50, 51]. Though MLCK and ROCK have the same phosphorylation sites, they have distinct roles in spatial regulation of MLC phosphorylation. Previous studies have demonstrated that ROCK-mediated direct phosphorylation of MLC and inhibition of MP lead to the assembly of stress fibers in the center of nonmuscle cells, while MLCK is involved in microfilament assembly in the periphery of the cells [52, 53]. Moreover, activated ROCK may promote disruption of E-cadherin-mediated AJs in epithelial cells by stimulating actomyosin contractility [54]. Another study demonstrated that disruption of AJs and TJs by inflammatory stimuli is induced by Rho and ROCK activation while attenuated by Rho and ROCK inhibition [55]. The activation of ROCK is Ca^2+^-independent which has been clearly shown in Ca^2+^-depleted epithelial cells [56]. The signaling pathways work as an intermediate step involved in the intracellular process triggered by extracellular cytokines, microbiota, or other chemicals. A number of proinflammatory cytokines induced epithelial breakdown is dependent on the activation of ROCK and/or MLCK signaling pathways.

It has been demonstrated that MLCK is required for maintenance of basal stress fibers in unstimulated cells but does not affect late stress fiber reorganization, morphological changes, or epithelial permeability, while ROCK is required for the maintenance of late stress fibers organization in TNF-*α* induced intestinal permeability [57]. TGF-*β*1 has been shown to play a role in the dissolution of TJs by Rho/ROCK signaling pathway [58]. IFN-*γ* induces endocytosis of epithelial AJC transmembrane proteins by Rho-ROCK-mediated contraction of perijunctional actomyosin cytoskeleton [56]. A recent study has found that apical bacterial internalization is regulated by IFN-*γ* induced MLCK-dependent brush border fanning associated with CD and celiac disease [59]. Several studies have demonstrated that TNF-*α* and other proinflammatory cytokines induce intestinal hyperpermeability by cytoplasmic-to-nuclear translocation of nuclear factor-kB (NF-kB). Therefore, NF-kB-regulated activation of MLCK promoter is the trigger for downstream increased expression of MLCK and subsequent opening of TJ barrier [21, 60–62]. Previous works have established that inflammatory cytokines are capable of independently reducing barrier function. However, more commonly, these cytokines induce barrier dysfunction synergistically [51].

## 5. Epithelial Barrier Breakdown and Gastrointestinal Disorders

MLCK- and ROCK- associated signaling pathways mediate actomyosin-dependent disruption of the epithelial barrier in different inflammatory settings. MLCK-mediated signaling pathway induces a modest contraction of perijunctional actomyosin belt and subsequently increases paracellular permeability without alterations in AJs structure, while ROCK-mediated signaling pathway is involved in the activation of the GEF-H1-Rho-ROCK pathway that leads to profound actomyosin contraction and TJs/AJs disassembly [63].

TJs are dynamic and are activated in different circumstances. Epithelial TJs and AJs have different sensitivity to different inflammatory cytokines. For example, TNF-*α* and INF-*γ* selectively disrupt TJs without affecting the AJ structure [64]. Alterations of the TJs in inflammatory conditions and disrupted epithelial barrier-induced mucosal immune activation are demonstrated in various gastrointestinal disorders. The detailed information of inflammatory cytokines and MLCK-/ROCK-mediated pathways observed in gastrointestinal disorders is listed in Tables 1 and 2.

## 6. Inflammatory Bowel Disease

IBD are relapsing and progressive inflammatory conditions that mainly affect the gastrointestinal tract [85]. There are two major forms: CD and UC [86]. Abnormal gut permeability has been identified in patients with IBD and also in some of their first-degree relatives [87]. The disruption of intestinal barrier induces exposure of luminal antigens to mucosal immune cells which subsequently leads to abnormal immune response [88]. Genetic studies have demonstrated an association between barrier integrity and epithelial regeneration-related genes in patients with IBD. Junction protein encoding genes like* CLDN1*,* CLDN2*, and* CDH1* are involved in the progression of disease activity in IBD [89, 90]. Moreover, it has been demonstrated that there is severe loss of occludin, ZO-1, and E-cadherin from AJC in the intestinal mucosa of patients with IBD [91].

As extracellular signals, all cytokines influence the expression of tight junction proteins resulting in interfering cellular junction structure and changing of intestinal integrity via Rho kinase-mediated F-actin cytoskeleton regulation [88]. Previous studies have shown that TNF-*α* induces epithelial MLCK activation which is related to barrier dysfunction in IBD. Moreover, the magnitude of MLCK expression correlated strongly with active inflammation [45].

Furthermore, TNF antagonism (infliximab) has therapeutic efficacy for patients with IBD, indicating that proinflammatory cytokines participate in the pathogenesis of IBD [65, 66]. IFN-*γ* is also mainly involved in regulation of immune response, and the expression is elevated in the intestinal mucosa in patients with IBD [67]. Furthermore, IFN-*γ* induces cellular internalization of transmembrane TJ proteins by activating small GTPase RhoA and subsequently regulating ROCK expression [82, 92]. In addition, the expression of IL-1*β* is also elevated in intestinal mucosa in patients with IBD, which causes intestinal inflammation and intestinal permeability [21]. Another study suggested that the barrier defect induced by IL-1*β* is associated with MLCK expression and MLC phosphorylation [93].

## 7. Celiac Disease

Celiac disease is an autoimmune disease of small intestine in genetically susceptible individuals and characterized by gluten sensitivity [94]. Sixty to 80% of patients with celiac disease show increased intestinal permeability leading to inflammatory reaction induced by luminal gliadin fractions [95, 96]. Many structural and molecular changes of epithelial tight junctions have already been reported. In duodenal biopsies of patients with untreated celiac disease, the levels of claudin-2 and occludin are increased while levels of claudin-3 and claudin-4 are decreased [96]. Genes, such as* ACTB*,* GNAII*,* TJPI*, and* CRB3*, that encode tight junctions showed altered levels of expression in patients with active celiac disease [97]. Previous studies suggest that the pathogenesis of celiac disease is driven by heightened Th1-predominant immune response [75]. Gluten elicits a response in antigen-presenting cells (dendritic cells, macrophages) that activate intraepithelial lymphocytes (IELs) and intestinal epithelial cells [98]. Taken together, numerous proinflammatory cytokines are involved in celiac disease including IFN-*γ*, IL-18, IFN-*α*, and IL-21 [75]. Proinflammatory cytokines such as IFN-*γ* and TNF-*α* contribute to the development of mucosal lesions in the small intestine along with villous atrophy and crypt hyperplasia. For example, the activation of NF-kB by IFN-*γ* causes occludin to dissemble and results in increased paracellular permeability [61, 99].

In addition,* MYO9B* is a good candidate gene for therapy in celiac disease as its encoded protein is involved in early mucosal inflammatory response.* MYO9B*-encoded protein belongs to class IX myosin molecules, which contains a Rho-GTPase-activating domain and regulates Rho/ROCK-dependent remodeling of cytoskeleton and epithelial permeability [5, 83]. However, the involvement of ROCK/MLCK in the pathogenesis of celiac disease requires further exploration.

## 8. Irritable Bowel Syndrome

IBS is a common functional gastrointestinal disorder with undetermined etiology [100]. The pathogenic factors of IBS recently reported are impaired barrier function, low-grade mucosal inflammation, and changes in intestinal microbiota composition [101, 102]. Altered expression of tight junction proteins including ZO-1 and occludin is responsible for increased intestinal permeability, especially in IBS patients with diarrhea predominant symptoms [103]. Mucosal cytokine composition changes and penetration of mast cell mediators into the mucosa play important roles in the modulation of intestinal permeability [104–106]. For example, proinflammatory cytokine IFN-*γ* strongly increases gut permeability, while anti-inflammatory IL-10 protects against the disruption of the TJ barrier [107]. Significantly increased levels of INF-*γ* and decreased levels of IL-10 have been shown in the intestinal mucosa of patients with IBS and also in postinfectious IBS [76, 77]. IFN-*γ* also plays an important role in visceral hypersensitivity in patients with IBS. IFN-*γ* induces MLC phosphorylation which leads to the contraction of epithelial cell cytoskeleton. The opening of TJ allows exposure to intraluminal agents that activates immune cells and sensitizes sensory nerve terminals to mechanical stimuli [108]. Finally, TNF-*α* also plays a role in the pathogenesis of IBS. TNF-*α* increases epithelial leakage by a mechanism that involves reorganization of tight junctions and perijunctional actomyosin ring that require MLCK [78, 109]. Another study has shown that there are increased phosphorylation of MLC and delayed redistribution of ZO-1 in epithelial cells after mucosal exposure to IBS-D supernatants. This suggests that intestinal hyperpermeability in IBS is related to ROCK or MLCK pathway [84].

## 9. Functional Dyspepsia

FD is also a common functional gastrointestinal disorder affecting 20% of population worldwide with poorly understood pathophysiology [110]. Recent studies have provided evidence for the presence of low-grade inflammation in the duodenal mucosa [111, 112]. Patients with FD have been shown to have significantly higher levels of circulating TNF-*α*, IL-1*β*, and IL-10 compared with healthy controls, and the cytokines release correlated with symptom onset of abdominal pain, cramps, nausea, and vomiting [81]. The low-grade duodenal inflammation in FD is associated with tight junction breakdown and increased intestinal permeability. Expression of OCLN and ZO-1 in the duodenal mucosa has shown to be significantly depressed in patients with FD [27]. Although there is lack of current evidence that MLCK or ROCK is involved in hyperpermeability and immune activation, MLCK/ROCK signaling pathways are essential routes contributing to epithelial barrier breakdown. The mechanism of barrier dysfunction and immune response in FD need further investigation.

## 10. Summary

Gastrointestinal mucosal epithelium provides an important role by performing a barrier to luminal antigens and maintaining mucosal homeostasis. Epithelial breakdown is associated with a number of gastrointestinal diseases [78, 113, 114]. Regardless of whether breakdown of intestinal barrier is the initial cause or the result of injury that contributes to pathology, restoring barrier function remains a worthwhile therapy in a variety of intestinal and extraintestinal diseases [16, 115]. The importance of the TJ barrier is demonstrated in nmMLCK knockout mice which showed protection from systemic or luminal stressors [116, 117]. Furthermore, inflammatory cytokines antagonism may also play a role in disease restoration, as TNF-*α* antagonism (infliximab) has efficacy in reducing inflammation and restoring gut barrier in patients with CD [65]. Elucidating upstream mechanism and key factors may provide important clues to develop novel therapeutic interventions in patients with debilitating diseases. Although inhibition of intestinal epithelial nmMLCK or ROCK to specifically restore barrier function may be therapeutically attractive, such attempts may not be clinically suitable up to now. Since the catalytic domain of MLCK in epithelium and smooth muscle are identical and MLCK/ROCK pathways are involved in a number of cellular activities and are important in maintaining cellular homeostasis, inhibitors are likely leading to unacceptable toxicities.

However, further basic research is still needed to improve our understanding of these complex signaling pathways and the crosstalk between them. The dynamic characteristic of AJC, the involvement of specific molecules, and intracellular signaling pathways that regulate epithelial functions offer opportunity for development of drugs with more specific actions in patients with gastrointestinal disorders.

## Figures and Tables

**Table 1:** Summary of inflammatory cytokines involved in gastrointestinal intestinal disorders.

Diseases	Species	Inflammatory cytokines	Reference
IBD	Human	TNF-*α*	Suenaert et al., 2002 [65]
			Järnerot et al., 2005 [66]
	Human	IFN-*γ*	Niessner and Volk, 1995 [67]
			Haep et al., 2015 [68]
			Rismo et al., 2012 [69]
	Human	IL-10	Niessner and Volk, 1995 [67]
	Human	IL-17A	Liu et al. 2016, [70]
			Rismo et al., 2012 [69]
	Human	IL-1*β*	Winchester and Pepine, 2015 [71]
	Human	IL-8	Rodríguez-Perlvárez et al.,2012 [72]
	Human	IL-2	Zaidi et al., 2016 [73]
	Human	IL-13	Heller et al., 2005 [24]

Celiac disease	Human	IL-15	Koning [74]
	Human	IFN-*γ*	Schuppan et al., 2009 [75]
		IL-21	Schuppan et al., 2009 [75]

IBS	Human	IFN-*γ*	Barbaro et al., 2016 [76]
	Human	IL-10	Chen et al., 2012 [77]
	Human	TNF-*α*	Vivinus-Nébot et al., 2014 [78]
		IL-6	Seyedmirzaee et al., 2016 [79]
		Il-8	Seyedmirzaee et al., 2016 [79]
		IL-1*β*	Pike et al., 2015 [80]

FD	Human	TNF-*α*	Liebregts et al., 2011 [81]
		IL-10	Liebregts et al., 2011 [81]
		IL-1*β*	Liebregts et al., 2011 [81]

**Table 2:** Summary of ROCK and MLCK signaling pathways in gastrointestinal intestinal disorders with increased intestinal permeability.

Diseases	Species	Signaling pathway	Reference
IBD	Human	MLCK	Blair et al., 2006 [45]
	Human	ROCK	Segain et al., 2003 [82]

Celiac disease	Human	ROCK	Monsuur et al., [83]

IBS	Human and mice	MLCK or ROCK	Gecse et al., 2008 [84]

## References

[1] Shen L., Turner J. R. (2006). Role of epithelial cells in initiation and propagation of intestinal inflammation. Eliminating the static: tight junction dynamics exposed. *American Journal of Physiology—Gastrointestinal and Liver Physiology*.

[2] Tsukita S., Furuse M., Itoh M. (2001). Multifunctional strands in tight junctions. *Nature Reviews Molecular Cell Biology*.

[3] Gehren A. S., Rocha M. R., de Souza W. F., Morgado-Díaz J. A. (2015). Alterations of the apical junctional complex and actin cytoskeleton and their role in colorectal cancer progression. *Tissue Barriers*.

[4] Dejana E. (2004). Endothelial cell-cell junctions: happy together. *Nature Reviews Molecular Cell Biology*.

[5] Matter K., Balda M. S. (2003). Signalling to and from tight junctions. *Nature Reviews Molecular Cell Biology*.

[6] Mège R.-M., Gavard J., Lambert M. (2006). Regulation of cell-cell junctions by the cytoskeleton. *Current Opinion in Cell Biology*.

[7] Nusrat A., Turner J. R., Madara J. L. (2000). Molecular physiology and pathophysiology of tight junctions. IV. Regulation of tight junctions by extracellular stimuli: nutrients, cytokines, and immune cells. *American Journal of Physiology—Gastrointestinal and Liver Physiology*.

[8] Ivanov A. I., Hunt D., Utech M., Nusrat A., Parkos C. A. (2005). Differential roles for actin polymerization and a myosin II motor in assembly of the epithelial apical junctional complex. *Molecular Biology of the Cell*.

[9] Naydenov N. G., Feygin A., Wang D. (2016). Nonmuscle myosin IIA regulates intestinal epithelial barrier *in vivo* and plays a protective role during experimental colitis. *Scientific Reports*.

[10] Vicente-Manzanares M., Ma X., Adelstein R. S., Horwitz A. R. (2009). Non-muscle myosin II takes centre stage in cell adhesion and migration. *Nature Reviews Molecular Cell Biology*.

[11] Heissler S. M., Manstein D. J. (2013). Nonmuscle myosin-2: mix and match. *Cellular and Molecular Life Sciences*.

[12] Adelstein R. S. (1982). Calmodulin and the regulation of the actin-myosin interaction in smooth muscle and nonmuscle cells. *Cell*.

[13] Amano M., Ito M., Kimura K. (1996). Phosphorylation and activation of myosin by Rho-associated kinase (Rho-kinase). *The Journal of Biological Chemistry*.

[14] Komatsu S., Ikebe M. (2004). ZIP kinase is responsible for the phosphorylation of myosin II and necessary for cell motility in mammalian fibroblasts. *Journal of Cell Biology*.

[15] Yamashiro S., Totsukawa G., Yamakita Y. (2003). Citron kinase, a Rho-dependent kinase, induces di-phosphorylation of regulatory light chain of myosin II. *Molecular Biology of the Cell*.

[16] Zolotarevsky Y., Hecht G., Koutsouris A. (2002). A membrane-permeant peptide that inhibits MLC kinase restores barrier function in in vitro models of intestinal disease. *Gastroenterology*.

[17] Nusrat A., Giry M., Turner J. R. (1995). Rho protein regulates tight junctions and perijunctional actin organization in polarized epithelia. *Proceedings of the National Academy of Sciences of the United States of America*.

[18] Ma T. Y., Tran D., Hoa N., Nguyen D., Merryfield M., Tarnawski A. (2000). Mechanism of extracellular calcium regulation of intestinal epithelial tight junction permeability: role of cytoskeletal involvement. *Microscopy Research and Technique*.

[19] Yang S., Yu M., Sun L. (2014). Interferon-*γ*-induced intestinal epithelial barrier dysfunction by NF-*κ*B/HIF-1*α* pathway. *Journal of Interferon & Cytokine Research*.

[20] Liu H., Wang P., Wang F.-J. (2011). An experimental study on intestinal epithelial barrier dysfunction induced by interferon-gamma and tumor necrosis factor-alpha. *Chinese Journal of Burns*.

[21] Al-Sadi R., Ye D., Dokladny K., Ma T. Y. (2008). Mechanism of IL-1*β*-induced increase in intestinal epithelial tight junction permeability. *The Journal of Immunology*.

[22] He F., Peng J., Deng X.-L. (2012). Mechanisms of tumor necrosis factor-alpha-induced leaks in intestine epithelial barrier. *Cytokine*.

[23] Suzuki T., Yoshinaga N., Tanabe S. (2011). Interleukin-6 (IL-6) regulates claudin-2 expression and tight junction permeability in intestinal epithelium. *Journal of Biological Chemistry*.

[24] Heller F., Florian P., Bojarski C. (2005). Interleukin-13 is the key effector Th2 cytokine in ulcerative colitis that affects epithelial tight junctions, apoptosis, and cell restitution. *Gastroenterology*.

[25] Clayburgh D. R., Shen L., Turner J. R. (2004). A porous defense: the leaky epithelial barrier in intestinal disease. *Laboratory Investigation*.

[26] Wilcz-Villega E., McClean S., O'Sullivan M. (2014). Reduced E-cadherin expression is associated with abdominal pain and symptom duration in a study of alternating and diarrhea predominant IBS. *Neurogastroenterology and Motility*.

[27] Vanheel H., Vicario M., Vanuytsel T. (2014). Impaired duodenal mucosal integrity and low-grade inflammation in functional dyspepsia. *Gut*.

[28] Hedin C. R., McCarthy N. E., Louis P. (2014). Altered intestinal microbiota and blood T cell phenotype are shared by patients with Crohn's disease and their unaffected siblings. *Gut*.

[29] Tolstanova G., Deng X., French S. W. (2012). Early endothelial damage and increased colonic vascular permeability in the development of experimental ulcerative colitis in rats and mice. *Laboratory Investigation*.

[30] Turner J. R., Rill B. K., Carlson S. L. (1997). Physiological regulation of epithelial tight junctions is associated with myosin light-chain phosphorylation. *The American Journal of Physiology*.

[31] Scott K. G. E., Meddings J. B., Kirk D. R., Lees-Miller S. P., Buret A. G. (2002). Intestinal infection with *Giardia* spp. reduces epithelial barrier function in a myosin light chain kinase-dependent fashion. *Gastroenterology*.

[32] Van Aelst L., D'Souza-Schorey C. (1997). Rho GTPases and signaling networks. *Genes & Development*.

[33] Bishop A. L., Hall A. (2000). Rho GTPases and their effector proteins. *The Biochemical Journal*.

[34] Rossman K. L., Der C. J., Sondek J. (2005). GEF means go: turning on RHO GTPases with guanine nucleotide-exchange factors. *Nature Reviews Molecular Cell Biology*.

[35] Ngok S. P., Lin W.-H., Anastasiadis P. Z. (2014). Establishment of epithelial polarity—GEF who's minding the GAP?. *Journal of Cell Science*.

[36] Riento K., Ridley A. J. (2003). Rocks: multifunctional kinases in cell behaviour. *Nature Reviews Molecular Cell Biology*.

[37] Schmandke A., Schmandke A., Strittmatter S. M. (2007). Reviews: ROCK and Rho: biochemistry and neuronal functions of Rho-associated protein kinases. *Neuroscientist*.

[38] Walsh S. V., Hopkins A. M., Chen J., Narumiya S., Parkos C. A., Nusrat A. (2001). Rho kinase regulates tight junction function and is necessary for tight junction assembly in polarized intestinal epithelia. *Gastroenterology*.

[39] Lu Q.-Y., Chen W., Lu L., Zheng Z., Xu X. (2014). Involvement of RhoA/ROCK1 signaling pathway in hyperglycemia-induced microvascular endothelial dysfunction in diabetic retinopathy. *International Journal of Clinical and Experimental Pathology*.

[40] Maekawa M., Ishizaki T., Boku S. (1999). Signaling from Rho to the actin cytoskeleton through protein kinases ROCK and LIM-kinase. *Science*.

[41] Turner J. R. (2009). Intestinal mucosal barrier function in health and disease. *Nature Reviews Immunology*.

[42] Verin A. D., Lazar V., Torry R. J., Labarrere C. A., Patterson C. E., Garcia J. G. N. (1998). Expression of a novel high molecular-weight myosin light chain kinase in endothelium. *American Journal of Respiratory Cell and Molecular Biology*.

[43] Clayburgh D. R., Rosen S., Witkowski E. D. (2004). A differentiation-dependent splice variant of myosin light chain kinase, MLCK1, regulates epithelial tight junction permeability. *The Journal of Biological Chemistry*.

[44] Zahs A., Bird M. D., Ramirez L., Turner J. R., Choudhry M. A., Kovacs E. J. (2012). Inhibition of long myosin light-chain kinase activation alleviates intestinal damage after binge ethanol exposure and burn injury. *American Journal of Physiology—Gastrointestinal and Liver Physiology*.

[45] Blair S. A., Kane S. V., Clayburgh D. R., Turner J. R. (2006). Epithelial myosin light chain kinase expression and activity are upregulated in inflammatory bowel disease. *Laboratory Investigation*.

[46] Chen C., Wang P., Su Q., Wang S., Wang F. (2012). Myosin light chain kinase mediates intestinal barrier disruption following burn injury. *PLoS ONE*.

[47] Martínez C., Lobo B., Pigrau M. (2013). Diarrhoea-predominant irritable bowel syndrome: an organic disorder with structural abnormalities in the jejunal epithelial barrier. *Gut*.

[48] Jou T.-S., Schneeberger E. E., Nelson W. J. (1998). Structural and functional regulation of tight junctions by RhoA and Rac1 small GTPases. *Journal of Cell Biology*.

[49] Mankertz J., Tavalali S., Schmitz H., Mankertz A., Riecken E. O., Fromm M. (2000). Expression from the human occludin promoter is affected by tumor necrosis factor alpha and interferon gamma. *Journal of Cell Science*.

[50] Benais-Pont G., Punn A., Flores-Maldonado C. (2003). Identification of a tight junction-associated guanine nucleotide exchange factor that activates Rho and regulates paracellular permeability. *Journal of Cell Biology*.

[51] Wang F., Graham W. V., Wang Y., Witkowski E. D., Schwarz B. T., Turner J. R. (2005). Interferon-*γ* and tumor necrosis factor-*α* synergize to induce intestinal epithelial barrier dysfunction by up-regulating myosin light chain kinase expression. *The American Journal of Pathology*.

[52] Totsukawa G., Yamakita Y., Yamashiro S., Hartshorne D. J., Sasaki Y., Matsumura F. (2000). Distinct roles of ROCK (Rho-kinase) and MLCK in spatial regulation of MLC phosphorylation for assembly of stress fibers and focal adhesions in 3T3 fibroblasts. *The Journal of Cell Biology*.

[53] Chrzanowska-Wodnicka M., Burridge K. (1996). Rho-stimulated contractility drives the formation of stress fibers and focal adhesions. *Journal of Cell Biology*.

[54] Sahai E., Marshall C. J. (2002). ROCK and Dia have opposing effects on adherens junctions downstream of Rho. *Nature Cell Biology*.

[55] Ivanov A. I., Samarin S. N., Bachar M., Parkos C. A., Nusrat A. (2009). Protein kinase C activation disrupts epithelial apical junctions via ROCK-II dependent stimulation of actomyosin contractility. *BMC Cell Biology*.

[56] Samarin S. N., Ivanov A. I., Flatau G., Parkos C. A., Nusrat A. (2007). Rho/Rho-associated kinase-II signaling mediates disassembly of epithelial apical junctions. *Molecular Biology of the Cell*.

[57] McKenzie J. A. G., Ridley A. J. (2007). Roles of Rho/ROCK and MLCK in TNF-*α*-induced changes in endothelial morphology and permeability. *Journal of Cellular Physiology*.

[58] Zhang K., Zhang H., Xiang H. (2013). TGF-*β*1 induces the dissolution of tight junctions in human renal proximal tubular cells: role of the RhoA/ROCK signaling pathway. *International Journal of Molecular Medicine*.

[59] Wu L.-L., Peng W.-H., Kuo W.-T. (2014). Commensal bacterial endocytosis in epithelial cells is dependent on myosin light chain kinase-activated brush border fanning by interferon-*γ*. *The American Journal of Pathology*.

[60] Ma T. Y., Boivin M. A., Ye D., Pedram A., Said H. M. (2005). Mechanism of TNF-*α* modulation of Caco-2 intestinal epithelial tight junction barrier: role of myosin light-chain kinase protein expression. *American Journal of Physiology—Gastrointestinal and Liver Physiology*.

[61] Thomas K. E., Sapone A., Fasano A., Vogel S. N. (2006). Gliadin stimulation of murine macrophage inflammatory gene expression and intestinal permeability are MyD88-dependent: role of the innate immune response in Celiac disease. *The Journal of Immunology*.

[62] Ye D., Ma I., Ma T. Y. (2006). Molecular mechanism of tumor necrosis factor-*α* modulation of intestinal epithelial tight junction barrier. *American Journal of Physiology—Gastrointestinal and Liver Physiology*.

[63] Ivanov A. I., Parkos C. A., Nusrat A. (2010). Cytoskeletal regulation of epithelial barrier function during inflammation. *The American Journal of Pathology*.

[64] Bruewer M., Luegering A., Kucharzik T. (2003). Proinflammatory cytokines disrupt epithelial barrier function by apoptosis-independent mechanisms. *The Journal of Immunology*.

[65] Suenaert P., Bulteel V., Lemmens L. (2002). Anti-tumor necrosis factor treatment restores the gut barrier in Crohn's disease. *The American Journal of Gastroenterology*.

[66] Järnerot G., Hertervig E., Friis-Liby I. (2005). Infliximab as rescue therapy in severe to moderately severe ulcerative colitis: a randomized, placebo-controlled study. *Gastroenterology*.

[67] Niessner M., Volk B. A. (1995). Altered Th1/Th2 cytokine profiles in the intestinal mucosa of patients with inflammatory bowel disease as assessed by quantitative reversed transcribed polymerase chain reaction (RT-PCR). *Clinical and Experimental Immunology*.

[68] Haep L., Britzen-Laurent N., Weber T. G. (2015). Interferon gamma counteracts the angiogenic switch and induces vascular permeability in dextran sulfate sodium colitis in mice. *Inflammatory Bowel Diseases*.

[69] Rismo R., Olsen T., Cui G., Christiansen I., Florholmen J., Goll R. (2012). Mucosal cytokine gene expression profiles as biomarkers of response to infliximab in ulcerative colitis. *Scandinavian Journal of Gastroenterology*.

[70] Liu Q. L., Huang L., Zhao Q. J., Li Q., He Z. (2016). Relationship between serum interleukin-17 level and inflammatory bowel disease. *Journal of Biological Regulators and Homeostatic Agents*.

[71] Winchester D. E., Pepine C. J. (2015). Angina treatments and prevention of cardiac events: an appraisal of the evidence. *European Heart Journal Supplements*.

[72] Rodríguez-Perlvárez M. L., García-Sánchez V., Villar-Pastor C. M. (2012). Role of serum cytokine profile in ulcerative colitis assessment. *Inflammatory Bowel Diseases*.

[73] Zaidi D., Bording-Jorgensen M., Huynh H. Q. (2016). Increased epithelial gap density in the non-inflamed duodenum of children with inflammatory bowel diseases. *Journal of Pediatric Gastroenterology and Nutrition*.

[74] Koning F. (2005). Celiac disease: caught between a rock and a hard place. *Gastroenterology*.

[75] Schuppan D., Junker Y., Barisani D. (2009). Celiac disease: from pathogenesis to novel therapies. *Gastroenterology*.

[76] Barbaro M. R., Di Sabatino A., Cremon C. (2016). Interferon-*γ* is increased in the gut of patients with irritable bowel syndrome and modulates serotonin metabolism. *American Journal of Physiology—Gastrointestinal and Liver Physiology*.

[77] Chen J., Zhang Y., Deng Z. (2012). Imbalanced shift of cytokine expression between T helper 1 and T helper 2 (Th1/Th2) in intestinal mucosa of patients with post-infectious irritable bowel syndrome. *BMC Gastroenterology*.

[78] Vivinus-Nébot M., Frin-Mathy G., Bzioueche H. (2014). Functional bowel symptoms in quiescent inflammatory bowel diseases: role of epithelial barrier disruption and low-grade inflammation. *Gut*.

[79] Seyedmirzaee S., Hayatbakhsh M. M., Ahmadi B. (2016). Serum immune biomarkers in irritable bowel syndrome. *Clinics and Research in Hepatology and Gastroenterology*.

[80] Pike B. L., Paden K. A., Alcala A. N. (2015). Immunological biomarkers in postinfectious irritable bowel syndrome. *Journal of Travel Medicine*.

[81] Liebregts T., Adam B., Bredack C. (2011). Small bowel homing T cells are associated with symptoms and delayed gastric emptying in functional dyspepsia. *The American Journal of Gastroenterology*.

[82] Segain J.-P., de la Blétière D. R., Sauzeau V. (2003). Rho kinase blockade prevents inflammation via nuclear factor *κ*B inhibition: evidence in Crohn's disease and experimental colitis. *Gastroenterology*.

[83] Monsuur A. J., de Bakker P. I. W., Alizadeh B. Z. (2005). Myosin IXB variant increases the risk of celiac disease and points toward a primary intestinal barrier defect. *Nature Genetics*.

[84] Gecse K., Róka R., Ferrier L. (2008). Increased faecal serine protease activity in diarrhoeic IBS patients: a colonic lumenal factor impairing colonic permeability and sensitivity. *Gut*.

[85] Kaser A., Zeissig S., Blumberg R. S. (2010). Inflammatory bowel disease. *Annual Review of Immunology*.

[86] Maloy K. J., Powrie F. (2011). Intestinal homeostasis and its breakdown in inflammatory bowel disease. *Nature*.

[87] Munkholm P., Langholz E., Hollander D. (1994). Intestinal permeability in patients with Crohn's disease and ulcerative colitis and their first degree relatives. *Gut*.

[88] Huang Y., Xiao S., Jiang Q. (2015). Role of Rho kinase signal pathway in inflammatory bowel disease. *International Journal of Clinical and Experimental Medicine*.

[89] Toedter G., Li K., Sague S. (2012). Genes associated with intestinal permeability in ulcerative colitis: changes in expression following infliximab therapy. *Inflammatory Bowel Diseases*.

[90] Muise A. M., Walters T. D., Glowacka W. K. (2009). Polymorphisms in E-cadherin (*CDH1*) result in a mislocalised cytoplasmic protein that is associated with Crohn's disease. *Gut*.

[91] Gassler N., Rohr C., Schneider A. (2001). Inflammatory bowel disease is associated with changes of enterocytic junctions. *American Journal of Physiology—Gastrointestinal and Liver Physiology*.

[92] Suzuki T. (2013). Regulation of intestinal epithelial permeability by tight junctions. *Cellular and Molecular Life Sciences*.

[93] Al-Sadi R., Guo S., Ye D. (2013). Mechanism of IL-1*β* modulation of intestinal epithelial barrier involves p38 kinase and activating transcription factor-2 activation. *Journal of Immunology*.

[94] Kang J. Y., Kang A. H. Y., Green A., Gwee K. A., Ho K. Y. (2013). Systematic review: worldwide variation in the frequency of coeliac disease and changes over time. *Alimentary Pharmacology and Therapeutics*.

[95] Sander G. R., Cummins A. G., Henshall T., Powell B. C. (2005). Rapid disruption of intestinal barrier function by gliadin involves altered expression of apical junctional proteins. *FEBS Letters*.

[96] Goswami P., Das P., Verma A. K. (2014). Are alterations of tight junctions at molecular and ultrastructural level different in duodenal biopsies of patients with celiac disease and Crohn's disease?. *Virchows Archiv*.

[97] Jauregi-Miguel A., Fernandez-Jimenez N., Irastorza I., Plaza-Izurieta L., Vitoria J. C., Bilbao J. R. (2014). Alteration of tight junction gene expression in celiac disease. *Journal of Pediatric Gastroenterology and Nutrition*.

[98] Tuckova L., Novotna J., Novak P. (2002). Activation of macrophages by gliadin fragments: isolation and characterization of active peptide. *Journal of Leukocyte Biology*.

[99] Helms S. (2005). Celiac disease and gluten-associated diseases. *Alternative Medicine Review*.

[100] Longstreth G. F., Thompson W. G., Chey W. D., Houghton L. A., Mearin F., Spiller R. C. (2006). Functional bowel disorders. *Gastroenterology*.

[101] Rajilić-Stojanović M., Biagi E., Heilig H. G. H. J. (2011). Global and deep molecular analysis of microbiota signatures in fecal samples from patients with irritable bowel syndrome. *Gastroenterology*.

[102] Turcotte J.-F., Kao D., Mah S. J. (2013). Breaks in the wall: increased gaps in the intestinal epithelium of irritable bowel syndrome patients identified by confocal laser endomicroscopy (with videos). *Gastrointestinal Endoscopy*.

[103] Bertiaux-Vandaële N., Youmba S. B., Belmonte L. (2011). The expression and the cellular distribution of the tight junction proteins are altered in irritable bowel syndrome patients with differences according to the disease subtype. *The American Journal of Gastroenterology*.

[104] Bashashati M., Rezaei N., Andrews C. N. (2012). Cytokines and irritable bowel syndrome: where do we stand?. *Cytokine*.

[105] Matricon J., Meleine M., Gelot A. (2012). Review article: associations between immune activation, intestinal permeability and the irritable bowel syndrome. *Alimentary Pharmacology & Therapeutics*.

[106] Lee H., Park J. H., Park D. I. (2013). Mucosal mast cell count is associated with intestinal permeability in patients with diarrhea predominant irritable bowel syndrome. *Journal of Neurogastroenterology and Motility*.

[107] Hu Y.-J., Wang Y.-D., Tan F.-Q., Yang W.-X. (2013). Regulation of paracellular permeability: factors and mechanisms. *Molecular Biology Reports*.

[108] Ait-Belgnaoui A., Bradesi S., Fioramonti J., Theodorou V., Bueno L. (2005). Acute stress-induced hypersensitivity to colonic distension depends upon increase in paracellular permeability: role of myosin light chain kinase. *Pain*.

[109] Clayburgh D. R., Barrett T. A., Tang Y. (2005). Epithelial myosin light chain kinase-dependent barrier dysfunction mediates T cell activation-induced diarrhea in vivo. *Journal of Clinical Investigation*.

[110] Ford A. C., Marwaha A., Sood R., Moayyedi P. (2015). Global prevalence of, and risk factors for, uninvestigated dyspepsia: a meta-analysis. *Gut*.

[111] Kindt S., Tertychnyy A., De Hertogh G., Geboes K., Tack J. (2009). Intestinal immune activation in presumed post-infectious functional dyspepsia. *Neurogastroenterology and Motility*.

[112] Futagami S., Shindo T., Kawagoe T. (2010). Migration of eosinophils and CCR2-/CD68-double positive cells into the duodenal mucosa of patients with postinfectious functional dyspepsia. *The American Journal of Gastroenterology*.

[113] Mankertz J., Schulzke J.-D. (2007). Altered permeability in inflammatory bowel disease: pathophysiology and clinical implications. *Current Opinion in Gastroenterology*.

[114] Mujagic Z., Ludidi S., Keszthelyi D. (2014). Small intestinal permeability is increased in diarrhoea predominant IBS, while alterations in gastroduodenal permeability in all IBS subtypes are largely attributable to confounders. *Alimentary Pharmacology & Therapeutics*.

[115] Takamura M., Sakamoto M., Genda T., Ichida T., Asakura H., Hirohashi S. (2001). Inhibition of intrahepatic metastasis of human hepatocellular carcinoma by Rho-associated protein kinase inhibitor Y-27632. *Hepatology*.

[116] Wainwright M. S., Rossi J., Schavocky J. (2003). Protein kinase involved in lung injury susceptibility: evidence from enzyme isoform genetic knockout and in vivo inhibitor treatment. *Proceedings of the National Academy of Sciences of the United States of America*.

[117] Reynoso R., Perrin R. M., Breslin J. W. (2007). A role for long chain myosin light chain kinase (MLCK-210) in microvascular hyperpermeability during severe burns. *Shock*.

